# An Edible Humidity Indicator That Responds to Changes in Humidity Mechanically

**DOI:** 10.1021/acsapm.3c00344

**Published:** 2023-06-05

**Authors:** Mengmeng Zhang, Abinaya Arunachalam, Hugo Perrin, Sevgi Polat, Jan Groenewold, Eduardo Mendes, Hüseyin Burak Eral

**Affiliations:** † Process & Energy Department, Delft University of Technology, Leeghwaterstraat 39, 2628 CB Delft, The Netherlands; ‡ Polymer Science, Zernike Institute for Advanced Materials, University of Groningen, Nijenborgh 4, Groningen 9747 AG, The Netherlands; ¶ Chemical Engineering Department, Faculty of Engineering, Marmara University, 34854 İstanbul, Turkey; § Van’t Hoff Laboratory, Physical Chemistry, University of Utrecht, Padualaan 8, 3584 CH Utrecht, The Netherlands; ∥ Guangdong Provincial Key Laboratory of Optical Information Materials and Technology, Institute of Electronic Paper Displays South China Academy of Advanced Optoelectronics, South China Normal University, Guangzhou 510006, P. R. China; ⊥ Chemical Engineering, Faculty of Applied Sciences, Delft University of Technology, Van der Maasweg 9, Delft, South Holland 2629 HZ, The Netherlands; # Van’t Hoff Laboratory, Physical Chemistry, University of Utrecht, Padualaan 8, 3584 CH Utrecht, The Netherlands

**Keywords:** Humidity indicator, Intelligent tag, Best-by date, Edible, Mechanical bending, Rolling, Caseinate film

## Abstract

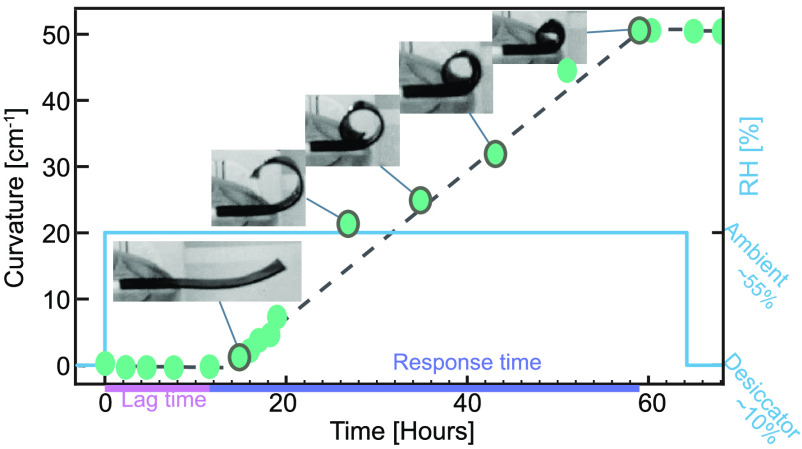

Elevated humidity levels in medical, food, and pharmaceutical products may reduce the products’ shelf life, trigger bacterial growth, and even lead to complete spoilage. In this study, we report a humidity indicator that mechanically bends and rolls itself irreversibly upon exposure to high humidity conditions. The indicator is made of two food-grade polymer films with distinct ratios of a milk protein, casein, and a plasticizer, glycerol, that are physically attached to each other. Based on the thermogravimetric analysis and microstructural characterization, we hypothesize that the bending mechanism is a result of hygroscopic swelling and consequent counter diffusion of water and glycerol. Guided by this mechanism, we demonstrate that the rolling behavior, including response time and final curvature, can be tuned by the geometric dimensions of the indicator. As the proposed indicator is made of food-grade ingredients, it can be placed directly in contact with perishable products to report exposure to undesirable humidity inside the package, without the risk of contaminating the product or causing oral toxicity in case of accidental digestion, features that commercial inedible electronic and chemo-chromatic sensors cannot provide presently.

## Introduction

The best-by date is mandatory and printed on packages of perishable products. Yet, it does not provide dynamic information on product safety prior to consumption. For instance, several products past these dates are usually safe to eat depending on consumer handling practices.^1^ Meanwhile, perishable products can also spoil before the reported best-by dates under improper storage conditions. Approximately 10% of food waste is a consequence of misinterpretation of the best-by date,^2^ resulting in considerable environmental and economic costs. Therefore, intelligent tags that can dynamically report on the storage environment are considered efficient to address these issues in food safety.

Intelligent tags are designed to monitor the storage environment of perishable products in a cost-efficient manner.^3^ Macroscopic size intelligent tags such as temperature tags^4−9^ and gas tags^10−14^ have been widely studied and are constantly reported in recent years. In addition to macroscopic size intelligent tags, microparticles^15^ that can report changes in pH^16^ or carry encoded information^17^ and respond to external stimuli^18^ have also been reported.

Detection of humidity is often overlooked in the research field of intelligent tags despite its relevance in the storage of perishable products. Condensation of moisture on perishables can lead to hastened deterioration, bacterial growth, and reduction in their estimated shelf life. Some common undesirable effects of excess humidity in food include caking, stickiness, lumping, crumbling, and molding.^19^ Medicines formulated as tablets and powders can also disintegrate or cake by absorbing excess moisture.^20^ Furthermore, medicines can deteriorate or exhibit undesirable chemical reactions, leading to reduced drug potency in the presence of excess moisture.

Research on humidity tags remains sparse to date despite the importance of humidity control as discussed above. The majority of reported humidity tags are chemo-chromatic; hence, they respond to changes in humidity by changing their color^12,21,22^ while other examples by virtue of geometric deformation have also been proposed.^23,24^

Thompson et al.^21^ proposed a chemo-chromatic tag using cobalt chloride to estimate the water activity of dried products. The easy hydration and dehydration reaction between CoCl_2_ and CoCl_2_·6H_2_O induces a color change, thus indicating a humidity shift. However, direct contact with consumable products was not advised due to the fact that chronic ingestion of cobalt compounds has been widely reported to cause serious health problems.^25^ Therefore, users are recommended to place this tag on the outside of the packaging, which introduces the risk of reading the ambient humidity instead of the true condition experienced by the target products. To overcome this, Bridgeman et al.^26^ proposed a hygroscopic composite tag of less toxic chemicals, i.e., sodium borohydride and DenimBlu30 dye. In this tag, DenimBlu30 dye functions as a redox indicator and evolves from yellow to blue after absorbing water, hence reporting the humidity increase. However, direct contact is still not recommended due to toxicity concerns.

Abandoning chemical reactions, Snyder et al.^24^ presented a tailored shape memory polymer of which the glass transition temperature can be driven lower to the operational temperature by vapor pressure. The absorbed water drives this transition by increasing the free volume and the mobility of polymer chains. Therefore, the initial rigid polymer develops into a flexible elastomeric state over time under vapor. However, the reversibility of this polymer only endows an instant humidity value and not the moisture exposure history.

Ionic liquids (ILs) have gained attention in recent years as they can be immobilized in polymer or metal–organic frameworks, enabling the creation of flexible composites that exhibit high sensitivity, fast response, and wearability as humidity sensors.^27−31^ A noteworthy study by Esteves et al.^28^ focused on gelatin/IL-based formulations, functioning as electrical and optical sensors; the formulations can be tailored to respond to humidity and detect volatile organic compounds under dry and humid conditions.

Despite the recent progress in intelligent tags, a biocompatible humidity indicator that can be placed in direct contact with food and medicine without contamination risk has not yet been reported in the literature to the best of our knowledge. The biocompatibility of the ionic liquids is not discussed in the above studies; it is quite promising to design fully biocompatible IL humidity sensors. Nevertheless, reading IL-based sensors requires the use of electrical devices due to the chemisorption of water molecules on the ILs which enhances its ionic conductivity and capacitance. However, this increases the cost of producing humidity tags and has environmental implications as the materials used in fabricating these electrical devices are typically plastics and metals. In addition, the indicators discussed above,^21,22,24,28^ unfortunately, exhibit reversible changes. Since a perishable product exposed to high humidity conditions still shows deterioration or bacterial growth even if the humidity is brought back to the recommended level, it is impractical to interpret spoilage accurately using reversible indicators. Therefore, a food-grade, edible, nontoxic, and irreversible humidity indicator with a tunable response time that matches the spoilage course is desirable.

In this study, we report an edible humidity indicator (EHI) made of food-grade ingredients that responds to undesired humidity exposure by irreversibly deforming and rolling on itself. The indicator is made of two food-grade polymer films with distinct protein, caseinate, to plasticizer, glycerol, ratios. Caseinate, originating from bovine milk, has been repeatably reported as a promising biodegradable packaging alternative in recent years.^32^ Based on the thermogravimetric analysis, we hypothesize that the bending mechanism is a result of hygroscopic swelling and consequent counter diffusion of glycerol and water. Leveraging our understanding of the bending mechanism, we demonstrated that EHI’s lag time, response time, and bending curvature can be tuned to expand its application scope. Overall, the developed indicator shows the potential to irreversibly detect undesired humidity exposure. The humidity indicator’s versatility is attributed to its ability to tune the lag time and response time by changing the layer thickness, making it suitable for applications where humidity changes occur gradually or infrequently over an extended period. The demonstrated response time is slow for applications requiring real-time or near-real time response. The indicator’s deformation in response to humidity makes it also suitable for use as a humidity-activated actuator, particularly in situations where low humidity levels need to be maintained. Moreover, thanks to its food-grade hence edible ingredients, the EHI has two distinct advantages compared to commercial nonedible electronic and chemo-chromatic sensors. First, the EHI can be placed in direct contact with perishable products; hence, it can report on the humidity level inside the packaging without any risk of contaminating the product. Second, in case the EHI is accidentally ingested, for instance, by infants or children, it does not pose any risk of oral toxicity.

## Results and Discussion

### Fabrication and Demonstration

**Figure 1:**
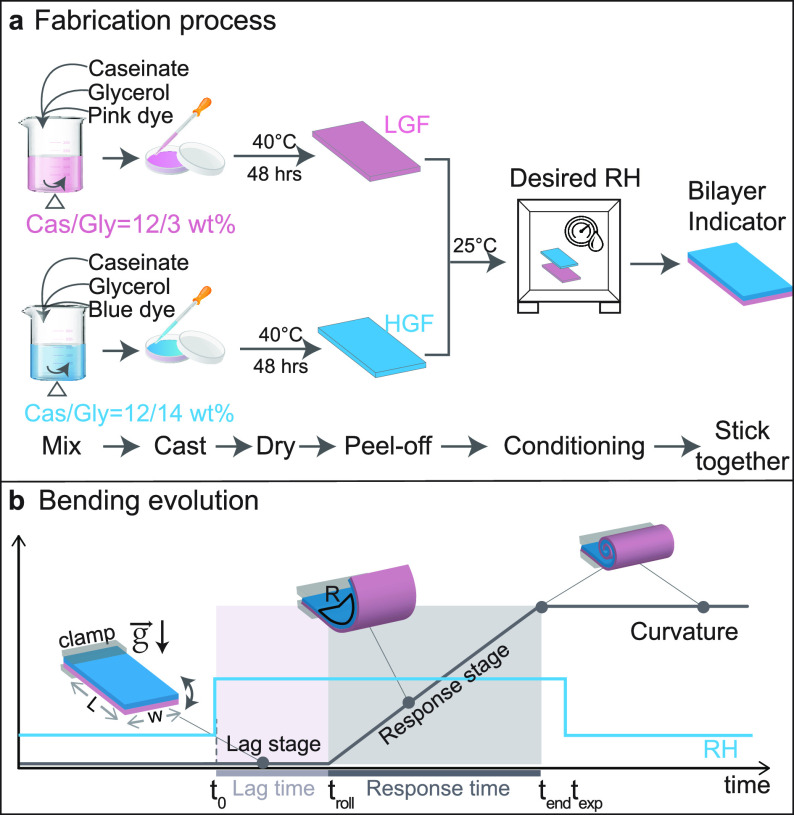
(a) Schematic representation of the fabrication process of the edible humidity indicator (EHI). (b) The flat EHI bends and rolls on itself to report humidity exposure. The bending evolution is irreversible and exhibits two stages including *Lag stage* and *Response stage*, which is schematically shown in the zoom plot (bottom). Figure not drawn to scale.

The fabrication process consists of several simple steps (Figure 1a) and is detailed in Fabrication of the EHI. In a nutshell, aqueous solutions containing two distinct ratios of caseinate and glycerol are evaporated to form films. To distinguish high glycerol content film (HGF, blue) and low glycerol content film (LGF, pink), blue and pink food dyes are added to the solutions prior to the evaporation step, respectively. Upon drying, the caseinate and glycerol solutions form a film. Due to the higher water affinity of glycerol, the HGF tends to absorb more water and swells more (Figures S1 and S2). The films are conditioned by storing them in desired humidity over 24 h. The conditioned films are then attached to each other leveraging the intrinsic sticky nature of films to fabricate bilayer EHIs.

To avoid potential oral toxicity issues, EHIs are made of only food-grade ingredients, namely, caseinate, glycerol, and water. Caseinate is proposed as biodegradable packaging material^33^ not only due to its attributes such as biocompatibility, biodegradability, and wide availability but also because of its random coil structure with the ability to form electrostatic, hydrophobic, and hydrogen bonds. Meanwhile, the casein film on its own is brittle and hence not easy to handle. To improve this, varying amounts of food-grade plasticizer glycerol are added to each layer. To ensure that EHIs are made of entirely nontoxic ingredients, no glue is adopted in the *stick-together* step. Perhaps due to caseinate’s self-healing ability, which rises from its random coil nature and ability to form weak intermolecular interactions within polymeric networks, i.e., electrostatic, hydrophobic, and hydrogen bonds as mentioned above,^34^ the single caseinate films can attach to each other and form a stable bilayer film. A scanning electron microscope image (Figure S3) shows that the gap between the two layers is negligible. In general, this composite bilayer contains only edible ingredients, which avoid any safety concern about direct contact with edible products.

#### Bending Evolution

The EHI response to accidental humidity exposure is illustrated as bending curvature development (Figure 1b). Moreover, the curvature development and macroshape of the EHI are detailed as well. The curvature is defined as the inverse radius of bending (*R*) as illustrated. Prior to humidity exposure, the EHI keeps its flat shape for a relatively long time (at least 15 days under 10% RH, Figure S4). We observed two distinct regimes, a *Lag stage* where the EHI absorbs water from the environment with no significant bending followed by a *Response stage* where the EHI deforms and gradually rolls on itself. After that, the EHI does not revert back to its initial shape when the humidity level is brought down. We refer to this experimental observation as the irreversible bending of the EHI, a feature that is essential for detecting and reporting humidity exposure.

To be more specific, we define *Lag stage* as the stage from *t*_0_ to *t*_roll_, where *t*_0_ is the moment the EHI is exposed to a high humidity level while *t*_roll_ is the moment the EHI starts to roll (see Figure 1b). In this *Lag stage*, the EHI commences to bend slightly and the direction of this minute bending changes multiple times, yet the curvature is negligible compared to hte *Response stage*. From *t*_roll_ on, the EHI enters into the *Response stage* where a pronounced bending is observed with a bending direction toward HGF. In other words, HGF remains inside the rolled structure. The curvature of the bending increases over time, and the EHI can roll on itself to form multiple rolls. After *t*_end_, the EHI holds its shape even when the humidity is brought back to the initial RH levels after days.

The reported bending and consequent rolling behavior are observed in other systems as a result of a mismatch in layer properties.^35^ Especially, the rolling phenomenon in the hydrogel system induced by the hygroscopic swelling differential between the two layers has been reported and leveraged in the design of hygroscopic bilayer structures.^36−41^ In these bilayer structures, one layer expands more and induces compressive stress on the other layer. Consequently, the hydrogel bilayer structure bends toward the layer with a lower swelling ratio. In other words, when these hydrogel bilayers bend and roll upon humidity exposure, less hygroscopic (less water-absorbing) film remains inside the rolled structure. We refer to this bending direction as rolling onto the less hygroscopic film.

Surprisingly, such a bending direction is not observed in our study. EHI sways back and forth slowly at the early stages of the exposure; it eventually bends toward and rolls onto HGF (more hygroscopic film, blue) regardless of RH value, EHI composition, or geometry. In other words, HGF (blue) remains inside the rolled structure and LGF (pink) remains outside (Figure 1b). This is intriguing since HGF has a higher water adsorption ability and a higher swellability than LGF (see Figures S1 and S2). If the bending mechanism of EHIs was identical to the aforementioned hydrogel bilayer structures, bending toward LGF would be expected. As we observe a different bending direction from the hydrogels in the literature, the bending mechanism of the EHI can not be solely explained by the distinct water adsorption capacity of two layers attached together, namely, HGF and LGF. A different rolling mechanism is needed to explain this observation.

#### Demonstrations

**Figure 2:**
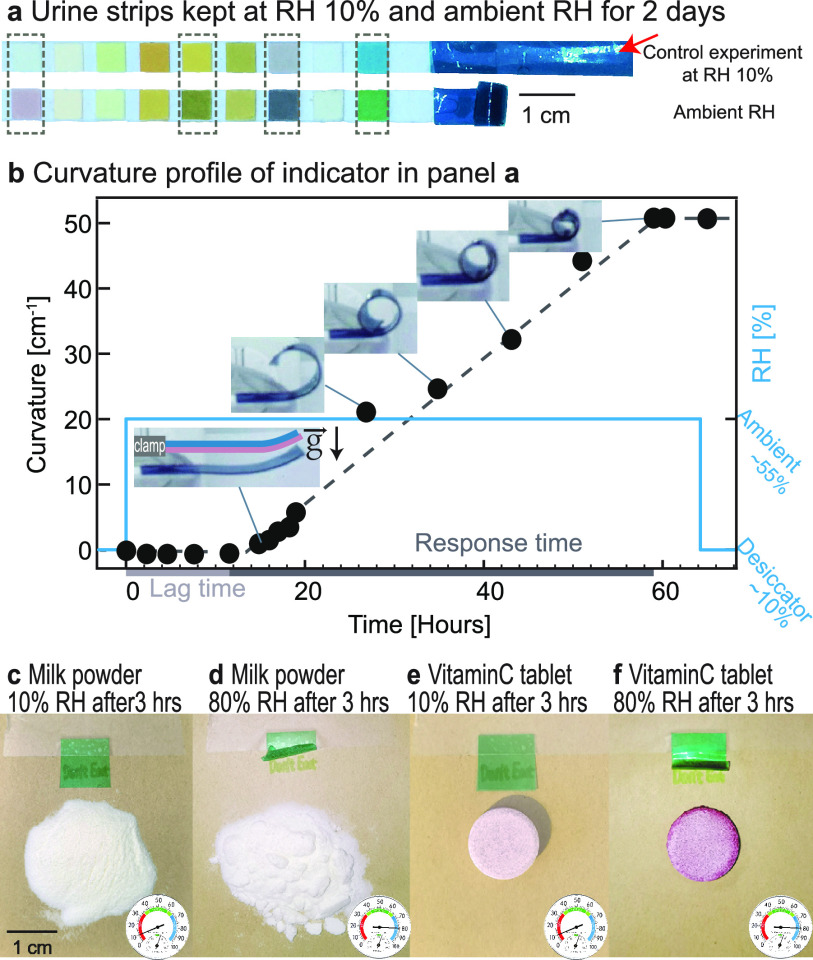
Demonstration on how the EHI (pointed out by red arrow) reports exposure to the elevated humidity by mechanical deformation. Panel (a) presents two EHI-attached urine strips stored under different humidity conditions after 2 days. After 2 days, the ability of the strips to fulfill their function is validated by identical artificial urine solutions. The strip at the top is the control sample stored at the recommended humidity level of 10% RH and gives the correct reading. However, the strip at the bottom, which is exposed to ambient conditions (fluctuating between 48% and 64% RH), gives a false reading evident from the color change distinct from the control (highlighted in gray). Panel (b) shows the curvature development of the EHI attached to the urine strip exposed to undesired ambient humidity conditions in panel (a) bottom. The dashed line is a guide for the eye. (c) Milk powder: 10% RH; 3 h in desiccator. (d) Milk powder: 80% RH; 3 h. (e) Vitamin C tablet: 10% RH; 3 h in desiccator. (f) Vitamin C tablet: 80% RH; 3 h. Moisture exposure diagnosed by a rolled EHI while there was no EHI bending in the control (desiccator-stored) experiments.

We showcase how the EHI can be implemented to monitor humidity levels of urine strips that need to be kept in a desiccated environment (Figure 2). The urine test strip aids in the fast screening of various diseases.^42^ Improper handling and storage of these test strips, especially due to humidity exposure, have been reported to give rise to incorrect diagnosis.^43^ We test whether the EHI can be used to detect urine test strips with compromised functionality due to humidity exposure (Figure 2).

For this test, we exposed urine strips with EHIs attached to lab humidity fluctuating between 48–64% throughout the experiment and monitored the bending of the EHI strips (Figure 2a, bottom). The humidity-exposed urine strip with an EHI attached is compared to the control sample kept at the recommended 10% RH at room temperature (Figure 2a, top). The control urine strip and exposed urine strip were afterward dipped in identical artificial urine solutions. The EHI exposed to ambient humidity rolls on itself, informing one of undesired exposure to high humidity, while the EHI in the control sample remained unaltered. Moreover, the control sample kept at 10% RH accurately measures the contents of artificial urine, but the exposed sample produces false readings. The bending is quantified by measuring the curvature (inverse radius, insets in Figure 2b) of the bent bilayer EHI extracted from time-lapse images of the EHI. Within a response time of 47 h, the curvature of the rolled EHI developed from 0 to 50 cm^–1^ after a lag time of approximately 13 h. After humidity exposure, the curvature remained even though the urine strip was put back into the desiccator where the RH was 10%. Furthermore, the EHI successfully diagnosed the exposure to humidity for two humidity-sensitive products, milk powder and Vitamin C tablet. The products and EHIs were kept at 80% RH for 3 h while the control sample was kept in a desiccator. The EHIs in humidity-exposed samples bent and rolled on themselves while the control sample remained flat (Figure 2c). To sum up, EHIs can identify a perishable product that has been once exposed to high humidity by irreversible bending and rolling. A video of the bending and rolling with more details is also provided in Movie S1. In addition, other EHI applications, i.e., pharmaceutical tablets and cashew nut, under 80% RH can be found (see Figure S5).

As different products degrade in distinct time scales, the broader applicability of EHI hinges on the tunability of the lag time and response time. The bending/rolling mechanism needs to be understood not just out of scientific interests but also from an engineering interest to benefit EHI design.

### Rolling Mechanism

#### TGA Result

As HGF (blue) has higher water absorption ability compared to LGF (pink), the EHI should have bent toward LGF if the bending mechanism was based solely on compressive stresses induced by the layer swelling more. Nevertheless, the observed bending direction contradicts this initial expectation. More interestingly, scanning electron microscopy images revealed that the LGF of the EHI bilayer elongated more than the HGF after 2 weeks of storage outside the desiccator at ambient humidity conditions (see Figure S3). Hence, we rationalize that a different physical mechanism should be in play.

**Figure 3:**
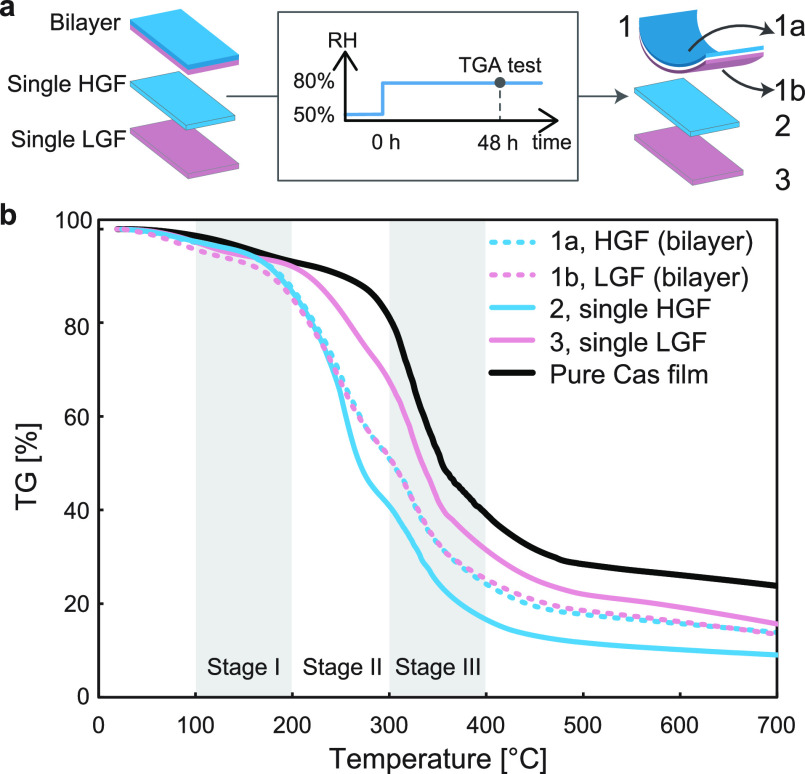
(a) The humidity exposure history and the referred name of various caseinate/glycerol films. (b) The TGA profiles of the tested films.

To shed light on the mechanism behind bending and consequent rolling of the EHI on itself, we conducted thermogravimetric analysis (TGA) on various films: a bilayer EHI(1), a single HGF(2), and a single LGF(3) (Figure 3a). Prior to TGA, the films were exposed to humidity. To accelerate the lag time, the conditioning of the single layers is at 50% RH instead of 10%. To amplify the water absorption and TGA results, we performed the test at 80% RH. After 48 h of exposure to 80% RH, the bilayer EHI(1) is separated manually by physically separating into HGF (bilayer, 1a) and LGF (bilayer, 1b). The TGA profiles of these films are obtained (Figure 3b). A pure caseinate film is also studied under the same condition to identify glycerol. Specific decomposition temperatures are also provided (Table S1).

TGA profiles show three distinct regimes. In stage I (100 to 200 °C), water evaporation induced a similar mass loss trend in all the samples. Stage III, from around 300 °C, is the decomposition of sodium caseinate, leaving 30% residue at 700 °C. In stage II, where the part of our interest lies, we found that films 1a and 1b show an identical trend after the humidity exposure, indicating that the compositions of these two films are identical. Therefore, it is rationalized to hypothesize that glycerol diffused from HGF to LGF after humidity exposure, considering that glycerol is the only chemical that decomposes within stage II.

#### Hypothesis for Bending and Rolling Mechanism

**Figure 4:**
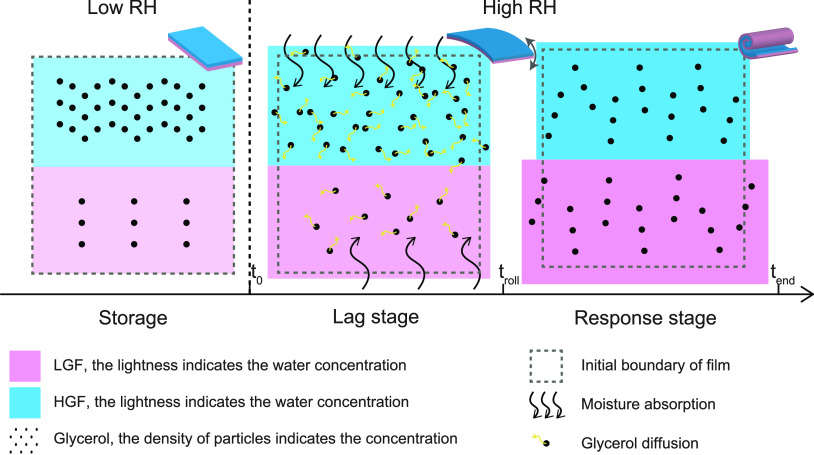
Proposed mechanism of rolling. Geometry and time are not drawn to scale.

Based on the TGA observations, we propose a rolling mechanism that involves the adsorption of condensed water from the environment and subsequently the counter diffusion of glycerol and water across the bilayer. The hypothesized mechanism (Figure 4) is illustrated to rationalize the macroscopic observations of bending and consequent rolling direction as well as observed lag and response time.

During the *Storage stage* (at *Low RH*), LGF and HGF absorb a limited amount of water. Glycerol is constrained in each layer at this stage since the caseinates are densely packed and the glycerol diffusion is considered extremely slow. Therefore, the EHI is capable of maintaining its flat shape for a relatively long time under 10% RH. In fact, as already mentioned, the EHI can maintain its shape for at least 15 days when subjected to low relative humidity (10% RH) (see Figure S4). This implies that the EHI is stable and reliable within our observation period and its shape will not be affected until it is exposed to a relatively high level of humidity. Shelf stability should be further investigated and adjusted based on the shelf life of the product, and EHIs will be implemented.

At *t*_0_ when humidity is introduced, both films start to absorb moisture and exhibit hygroscopic swelling. In this stage, which we term as *Lag stage*, we speculate that the films absorb more water and exhibit a volumetric change compared to the initial volume. As previously mentioned, the high concentration of glycerol in the HGF tends to bond more water, and there is a larger volume change in the HGF (Δ*V*_HGF_) compared to that of the LGF (Δ*V*_LGF_). On the macroscale, the bilayer first bends toward the LGF with the HGF as the outer layer and the LGF as the inner layer (Movie S1). However, since the network of the caseinate film is expanded by the absorbed moisture, the glycerol molecules from this moment can move from the HGF to LGF due to the concentration gradient. We hypothesize that moisture uptake and glycerol diffusion along with water diffusion lead to a competition in volume change between the LGF and the HGF (Δ*V*_LGF_ ≈ Δ*V*_HGF_). On the macroscale, we hypothesize the lag time observed is due to the time required for the condensed water to penetrate the glassy network.

In the *Response stage*, the bulk volume of each layer further expands; hence, the caseinate proteins are loosely packed, and the size of the pores within the caseinate network further grows. As a result, glycerol in this stage diffuses significantly from the HGF to the LGF. Furthermore, water absorbed by the HGF migrates to the LGF accompanied by glycerol diffusion. These two factors henceforth help the LGF eventually win the competition in the volumetric increase (Δ*V*_LGF_ ≫ Δ*V*_HGF_) and lead the bilayer to roll up with the LGF as the outer layer along with HGF as the inner^44^ layer on the macroscale.

Moreover, interface effects such as the formation of a thick skin layer due to enhanced evaporation at corners and at the interface may alter local microstructure in EHI. The altered crust skin may slow down the diffusion of water consequently time scale of bending.

After *Response stage*, the counter diffusion of glycerol and water reaches equilibrium, and the two layers become identical in composition as supported by TGA results. The rolling of the EHI on itself stops since there is no concentration gradient that can drive the dilatation. Similarly, since the bending and rolling are proposed to be driven by glycerol diffusion, the process cannot exhibit reversibility because there is no glycerol concentration difference in the equilibrium bilayer to provide the necessary driving force.

### Tunability

It is crucial to engineering the EHI response, namely, the lag time, response time, and final curvature of rolling, to match the characteristic time scale of decomposition/degradation of the labeled products. In this section, we will demonstrate that the EHI response can be tuned by simply adjusting the layer thickness and aspect ratio.

#### Tunability of Lag Time and Response Time

**Figure 5:**
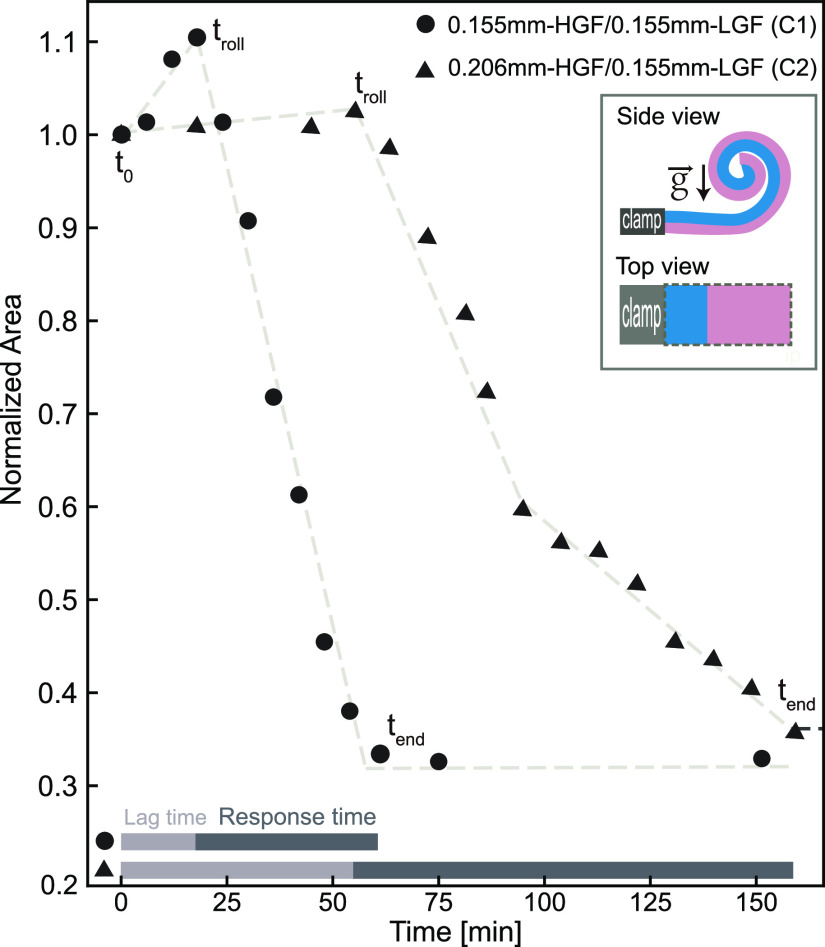
Normalized area (Projected area/Initial area) of EHIs as a function time for two representative EHIs with different layer thicknesses. The thickness of the films making up the EHIs are 0.155 mm-HGF/0.155 mm-LGF (C1, dots) and 0.206 mm-HGF/0.155 mm-LGF (C2, triangles), respectively. The films are conditioned at 50% RH as single layers for 24 h, stuck together, and tested at 80% RH. The measurement parameters are shown in the inset. Dashed lines are a guide for the eye.

The tunability of lag time and response time through layer thickness is showcased by two EHIs with different thicknesses, i.e., 0.155 mm-HGF/0.155 mm-LGF (Figure 5, C1, dots) and 0.206 mm-HGF/0.155 mm-LGF (Figure 5, C2, triangles). The single layers are conditioned at 50% RH for 24 h before being stuck together and tested at 80% RH. Under 80% RH, the EHI rolls on itself with a nearly constant curvature by forming a spiral at the end as sketched in the inset. For this reason, *Normalized Area*, i.e., Projected area/Initial area, instead of the curvature, is given to represent the rolling behavior. The projected area is highlighted by the gray dashed line (Figure 5).

The lag time of the thinner EHI combination (C1, dots, 0.155 mm-HGF/0.155 mm-LGF) is 25 min. It exhibits a linear rolling behavior in line with earlier presented results that is completed within 30 min. The rolled structure is maintained after 150 min. For the thicker combination (C2, triangles, 0.206 mm-HGF/0.155 mm-LGF), the lag time is 60 min; meanwhile, it takes around 90 min to complete rolling. By increasing around 30% of the HGF thickness, both the lag time and response time are doubled. We can attribute this longer operation time to two reasons. First, a thicker film spends more time reaching the swollen equilibrium state. Second, the thicker combination, due to its reduced flexibility,^36^ has a tendency to bend at a slower pace, resulting in a longer response time when compared to the thinner combination. Similar to C1, C2 also holds its final structure after *t*_end_ (data not shown in the figure). Both C1 and C2 show a normalized area increase during the lag stage. This increase in the area could be attributed to the films swelling when they absorb moisture, as we already discussed (Figure 4).

However, the device is considered versatile due to the ability to easily tune its lag time and response time through the layer thickness, making it suitable for various applications where humidity changes slowly or infrequently over a longer period of time, such as monitoring humidity trends in a climate-controlled environment such as storage room. The response time varies from 90 to 150 min in the showcased combinations. While this showcased response time may not be ideal for applications requiring a real-time response, it is suitable for warning users of slowly evolving humidity-triggered processes such as bacterial or mold growth which typically requires multiple hours to a few days based on environmental conditions and biological species.

#### Tunability of Final Curvature

**Figure 6:**
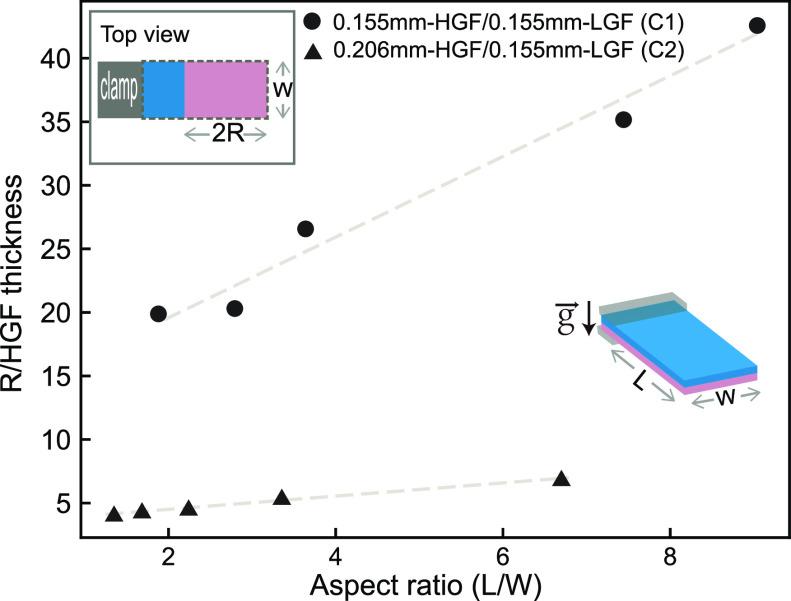
Final radius of the rolled EHI normalized by the thickness of the HGF layer as a function of aspect ratio for EHIs with different layer thickness combinations. The thickness combinations of the EHIs are 0.155 mm-HGF/0.155 mm-LGF (C1, dots) and 0.206 mm-HGF/0.155 mm-LGF (C2, triangles), respectively. The films are conditioned at 50% RH and tested at 80% RH. The measured parameters from the final rolled EHIs are schematically shown in the inset. The gray dashed line is the linear fitting.

The aspect ratio has already been consistently reported to influence the bending conformation (i.e., it dictates whether a bilayer structure rolls along the long or short edge) for other bilayer systems.^35,45−47^ However, contradicting results exist in the literature regarding the quantitative relationship between the aspect ratio and final bending curvature. Stoychev et al.,^35^ Wang et al.,^48^ and Abdolahi et al.^49^ found the bending curvature of the free-standing hydrogel bilayer is independent of the aspect ratio, which agrees with Timoshenko’s theory.^50^ However, Kim et al.^51^ observed a strong dependence between aspect ratio and bilayer bending. Moreover, multiple-rolling has been observed exclusively under the condition of a large aspect ratio by Alben et al.^45^ We already found that at 50% RH all our EHIs roll to a radius of around 2.5 mm regardless of aspect ratio (see Figure S6), where the rolling degree can be considered uniform within experimental limits. In this study, the relationship between the aspect ratio and the attained curvature of the rolled EHI is further investigated for EHIs conditioned at 50% RH and exposed to 80% RH. The results are summarized in Figure 6.

As the aspect ratio influences the bending conformation, to be more specific, bilayers will bend into a shape that minimizes resistance to bending in an energetically preferred way.^51^ In this study, we only focus on the final curvature the rolled EHI attains because bending conformation is less critical for humidity interpretation according to our observations. To eliminate certain conformations, the short edge of the EHI is attached to a solid object. When the short edge is fixed by a clamp, the EHI looks like a cantilever beam, and the bending occurs along the long edge.

EHIs of two distinct thicknesses (both C1 and C2) were prepared in various aspect ratios, conditioned under 50% RH, and exposed to 80% RH. The final shape of the rolled EHI is characterized by radius (*R*) as illustrated in the inset. We further normalize the radius with the thickness of the HGF of the EHI to ensure that we have a dimensionless graph (Figure 6). Interestingly, there is a clear linear correlation between *R*/*HGF thickness* and the aspect ratio *l*/*w*. The coefficient of determination (*R*^2^ ≈ 0.97 for C1 and *R*^2^ ≈ 0.99 for C2) indicates a reasonably strong, positive linear correlation between nondimensionalized *R* and aspect ratio. Since the length and the HGF layer thickness are fixed, while the width is varied in this set of tests, we can also put it in a more simple way; i.e., the final rolling radius is larger for smaller widths. It can be seen that C2 (the thicker combination, 0.206 mm-HGF/0.155 mm-LGF) overall shows a smaller *R*, which is within expectation considering that the larger total amount of glycerol transported from the HGF to the LGF can induce more pronounced swelling in the LGF in C2 compared to that in C1. Moreover, when we compare the two slopes of C1 and C2, it is evident from the observation that the aspect ratio exerts a more substantial effect on the final radius of the rolled bilayer EHI in thinner C1 compared to C2.

It is concluded that, in this caseinate/glycerol bilayer, the final size of the roll is influenced by the width of EHI under a higher humidity level, namely, 80% RH. A similar observation was also found in a micromachined polysilicon/chromium bilayer cantilever.^52^ At this moment, we can not offer a quantitative theory explaining this observation, yet we speculate that the torque generated by the clamp along the width possibly plays a role in this phenomenon. The reason that the aspect ratio only influences the radius of the final shape under 80% is unclear to us at the moment. As discussed previously, we hypothesize that the bending of the EHI is initiated by glycerol diffusion along with water migration. During exposure to humidity, the water and glycerol molecules diffuse between layers until they reach the equilibrium state. The volume of each layer is also altered dynamically during this process. Therefore, we rationalize that adoption of the Timoshenko’s theory or the modified equation from Reyssat and Mahadevan^50^ is not possible for EHIs conditioned at 50% RH and then exposed to 80% RH where diffusion occurs.

## Conclusion

We developed a caseinate/glycerol-based food-grade hence edible humidity indicator that informs one of undesired humidity exposure by irreversible mechanical bending. We characterized the rolling time and final geometry of the EHI when exposed to high humidity levels as a function of EHI thickness and aspect ratio, respectively. Moreover, the application of the EHI has been demonstrated with a humidity-sensitive medical test kit, pharmaceutical tablets, milk powder, etc. We proposed a rolling mechanism coupling hygroscopic swelling, glycerol diffusion, and water diffusion. This mechanism is substantiated by TGA and guides us to tune the lag time and response time by manipulating the layer thickness. Meanwhile, the final curvature of the rolled EHI is shown to depend on the aspect ratio under 80% RH. However, when EHI is applied to label products in real life, one should take temperature variations into account since it affects diffusion. This is not addressed in this study but is worth investigating in the future. Overall, the developed indicator shows the potential to detect perishable products exposed to unsuitable humidity levels by deforming irreversibly upon humidity exposure. The indicator’s ability to deform in reaction to humidity also renders it appropriate as a humidity-activated actuator, especially in scenarios where it is essential to uphold low humidity levels.

## Materials and Methods

### Fabrication of the EHI

The EHI is composed of two films containing distinct amounts of caseinate, glycerol, and water in each. We refer to the film with higher and lower glycerol ratios as high glycerol film (HGF) and low glycerol film (LGF), respectively. An illustration of the film production procedure is in Figure 1a and will be detailed as follows.

To prepare the film-forming mixtures, ultrapure water (Millipore Milli-Q IQ 7000 system, 18 mΩ cm) and glycerol (CAS 56-81-5, Boom B.V., 86%) were mixed and magnetically stirred at 600 rpm and 60 °C. To this solution, sodium caseinate (CAS 9005-46, Sigma-Aldrich) was gradually added over time intervals of 2–3 min to reach the desired caseinate/glycerol/water ratio, which is 12:14:100 for the HGF (dyed blue) and 12:3:100 for the LGF (dyed pink), respectively. The stirring rate was subsequently increased to 850 rpm for 60 min. Afterward, food dyes (Dr. Oetker) were added to distinguish the HGF and the LGF. The final mixtures were centrifuged at 7400 rpm for 12 min to remove any bubbles formed during the mixing process.

Film-forming mixtures were cast into plastic Petri dishes (9 cm in diameter, Sigma-Aldrich) and baked in the oven at 40 °C for 48 h. After gelation, the films were peeled off and stored in a desiccator (RH 10%). Films are conditioned for 24 h in an environmental chamber (BTL-433, ESPEC) under specific RH before the test.

Moreover, the thickness of the film was controlled by the amount of the mixture used and measured by a film thickness gauge (Heidenhain). For instance, a 12 mL mixture can obtain an HGF of 0.310 mm thick and an LGF of 0.155 mm thick.

### Protocol of Demonstrations

The utility of EHIs is demonstrated with a urinalysis reagent strip (InSight, Acon Laboratories). An EHI strip (0.155 mm-HGF and 0.155 mm-LGF) of 5 mm by 20 mm was cut and attached to the blank end of the urinalysis strip along the short side with Norland Optical adhesive that cures using ultraviolet light. All EHIs were conditioned under 10% before usage and then exposed to atmospheric humidity that simulated a consumer leaving the actual storage bottle open by accident. The test was conducted at a lab temperature that fluctuated around 23 °C. After a 48 h atmospheric humidity exposure, we kept the test urine strip back in the desiccator for 4 h to examine any reversibility. Following this, a urinalysis test was conducted on the model strip and compared with an original strip unexposed to atmospheric humidity. The preparation of artificial urine is the same as described in the literature.^53^

For visualization, the EHI response was recorded throughout the process in the form of a time-lapse with an action camera (4K/30FPS video resolution, 20 megapixels, and a 170-degree super wide-angle 6G fisheye lens, Apexcam) to visualize the process. To measure the curvature evolution, the recorded time-lapse film was extracted per certain frames. The radius (*R*) of the rolled EHIs was analyzed using ImageJ. The rolling curvature was calculated as 1/*R*.

### Thermogravimetric Analysis (TGA)

The thermal properties of the caseinate samples obtained in different glycerol concentrations were examined using thermogravimetric analysis (TGA) conducted on an SDTQ600 instrument. In each analysis, approximately 8.0 ± 0.5 mg of sample was placed in an aluminum oxide crucible and heated over temperatures ranging from 30 to 750 °C at a rate of 20 °C/min. A continuous nitrogen flow at a rate of 30 mL/min was supplied to the TGA to maintain an inert atmosphere.

### Thickness and Aspect Ratio Test

For the thickness test, two combinations were tested. Combination 1 (C1) was made of a 0.155 mm-thick HGF and a 0.155 mm-thick LGF while combination 2 (C2) was a 0.206 mm-thick HGF and a 0.155 mm-thick LGF. After conditioning separately under 50% RH for 24 h, the single-layer films were stuck together and immediately cut into rectangular strips. One end of the samples was fixed to a glass slide with water-proof tape. The ready-to-test EHIs are 15 mm in length. After that, the samples were transferred into the environmental chamber at 80% RH and a constant temperature of 25 °C. The EHI response was recorded in time-lapse from the top view to visualize the rolling process. Finally, pictures were extracted, and the projected area of the rolled circle was analyzed using ImageJ image analysis software. We use *Normalized Area* to represent the rolling in this test, which was calculated as Projected area/Initial area.

For the aspect ratio test, the conditioning and test procedure were the same as the thickness test as described above besides that samples were cut into rectangular strips of various lengths and widths. We only focus on the final geometries. Therefore, images were only captured after the rolling was completed. The radius (*R*) of the rolls was analyzed using ImageJ as well.

## Supporting Information Available

Moisture absorption and desorption performance of glycerol films; thickness increase of LGF and HGF; scanning electron microscopy (SEM) and X-ray diffraction (XRD) analyses; images of EHIs; normalized curvature of C1 as aspect ratio; specific decomposition temperatures of various EHI films (PDF)
Video of the curvature development of the indicator attached to a urine strip (MOV)

## Supplementary Material

ap3c00344_si_001.pdf

ap3c00344_si_002.mov
