# Supplementary material for: An Edible Humidity Indicator That Responds to Changes in Humidity Mechanically
Source: ACS Appl Polym Mater. 2023 Jun 5;5(7):4780–8. doi: 10.1021/acsapm.3c00344 (PMC10353009; doi:10.1021/acsapm.3c00344)
Supplement: Supplementary file 1 — ap3c00344_si_001.pdf [file ap3c00344_si_001.pdf]

Supporting Information
An edible humidity indicator that responds to changes in humidity mechanically

Mengmeng Zhang,[†] Abinaya Arunachalam,[†,‡] Hugo Perrin,[†] Sevgi Polat,[†,¶] Jan Groenewold,[§,‖] Eduardo Mendes,[⊥] and Hüseyin Burak Eral[*,†,#]

†*Process & Energy Department, Delft University of Technology, Leeghwaterstraat 39, 2628 CB Delft, The Netherlands*

‡*Polymer Science, Zernike Institute for Advanced Materials, University of Groningen, Nijenborgh 4, Groningen, 9747 AG The Netherlands*

¶*Chemical Engineering Department, Faculty of Engineering, Marmara University, 34854, İstanbul, Turkey*

§*Van't Hoff Laboratory,Physical Chemistry, University of Utrecht, Padualaan 8, Utrecht, 3584 CH, Utrecht, The Netherlands*

‖*Guangdong Provincial Key Laboratory of Optical Information Materials and Technology, Institute of Electronic Paper Displays South China Academy of Advanced Optoelectronics, South China Normal University, Guangzhou 510006 P. R. China*

⊥*Chemical Engineering, Faculty of Applied Sciences, Delft University of Technology, Van der Maasweg 9, Delft, 2629 HZ, South Holland The Netherlands*

#*Van't Hoff Laboratory, Physical Chemistry, University of Utrecht, Padualaan 8, Utrecht, 3584 CH, Utrecht, The Netherlands*

E-mail: h.b.eral@tudelft.nl

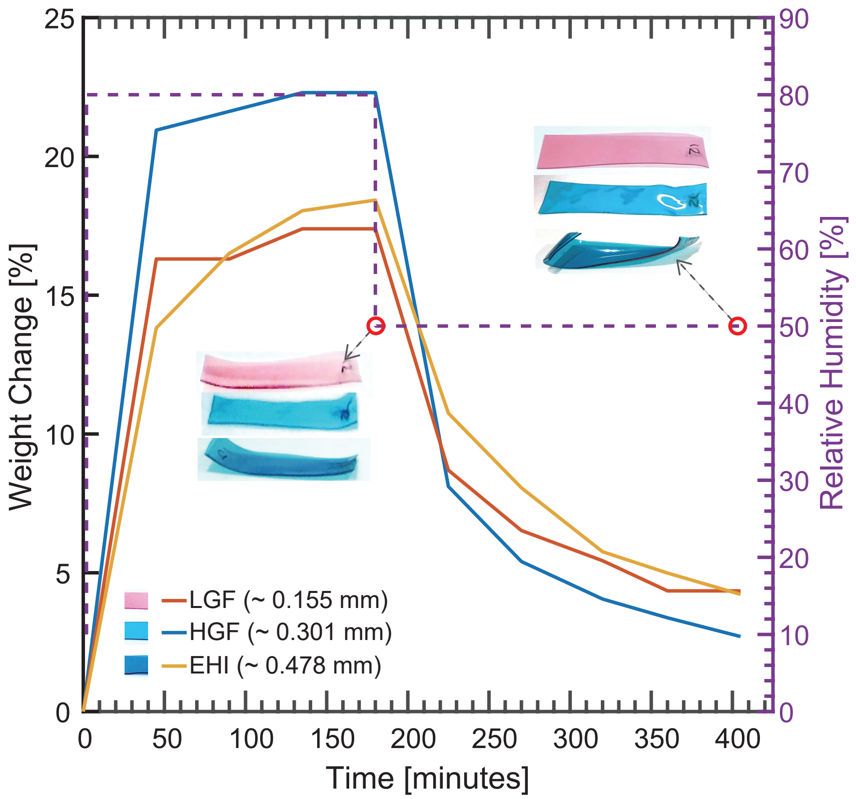

Figure S1. The moisture absorption and desorption performance of high glycerol film (HGF), low glycerol film (LGF), and a bilayer sensor composed of these two films (top) measured by the weight percentage change on the left Y-axis.

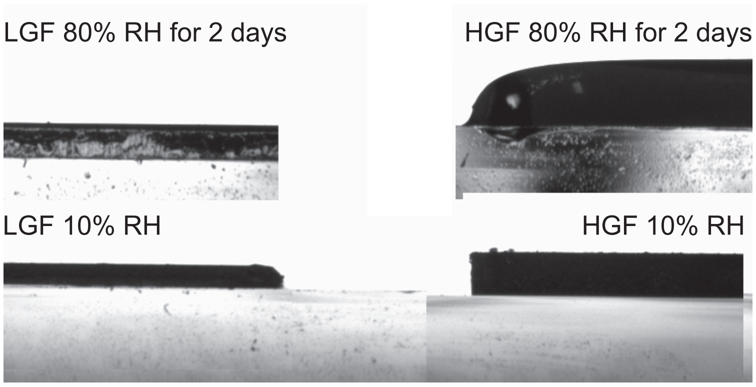

Figure S2. Thickness increase of LGF and HGF after exposure to 80% for 2 days.

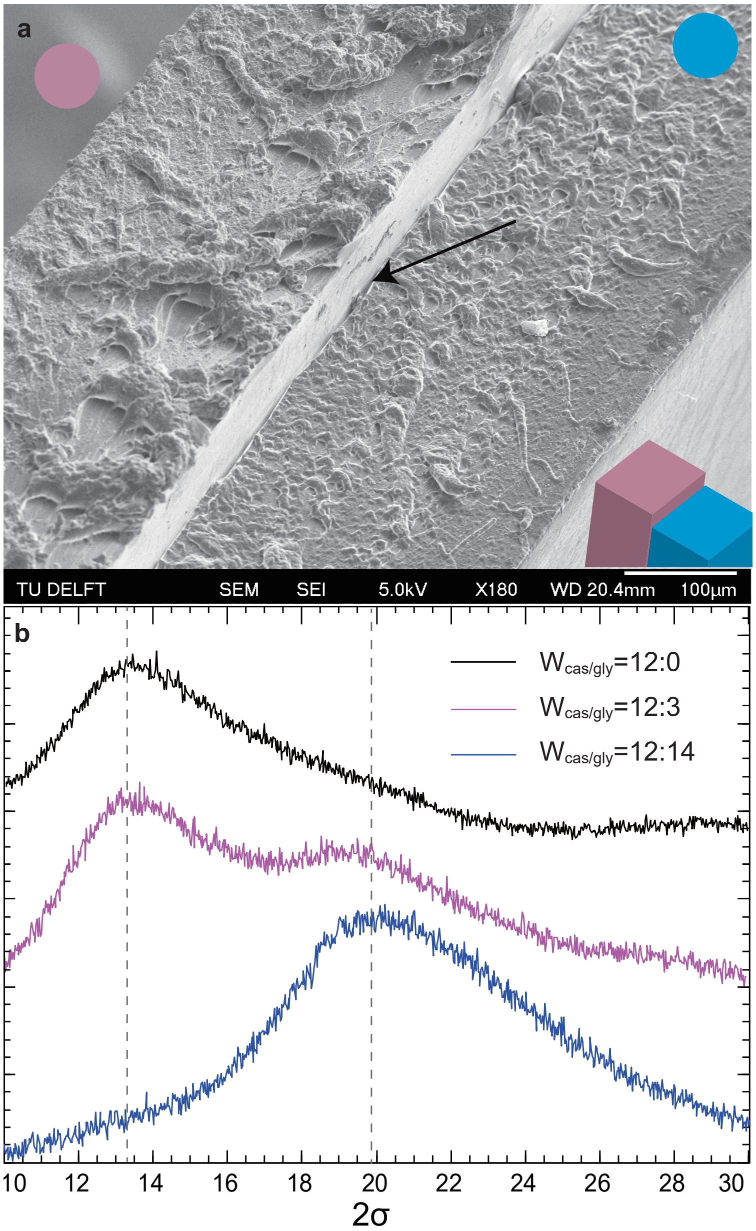

Figure S3. Scanning electron microscope (SEM) image of the bilayer (a) and XRD profiles of various single films (b).

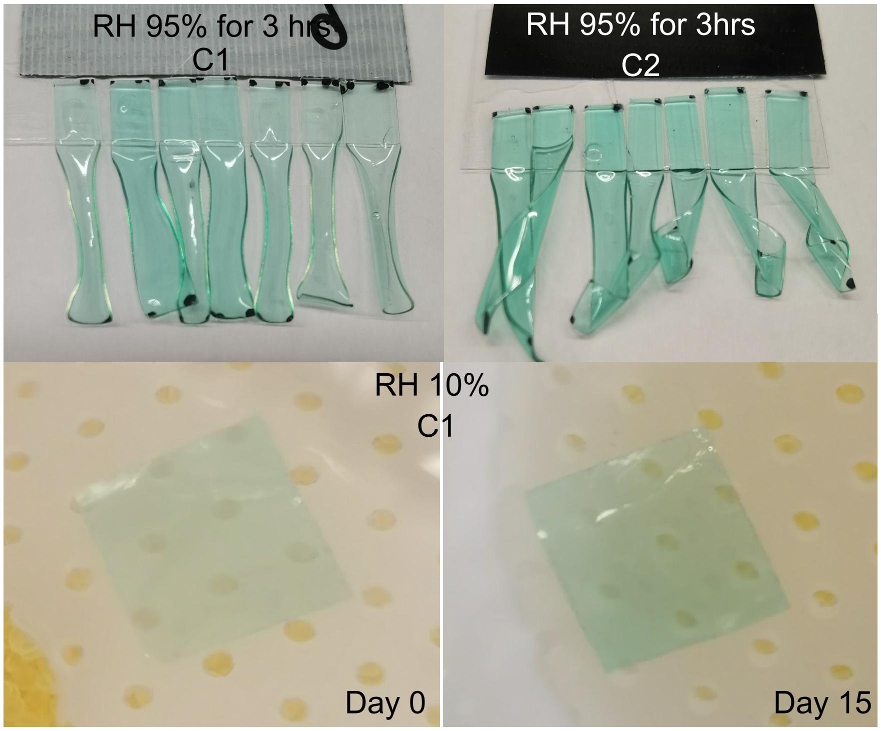

Figure S4. Images of EHIs bending under high RH (95%) and low RH (10%).

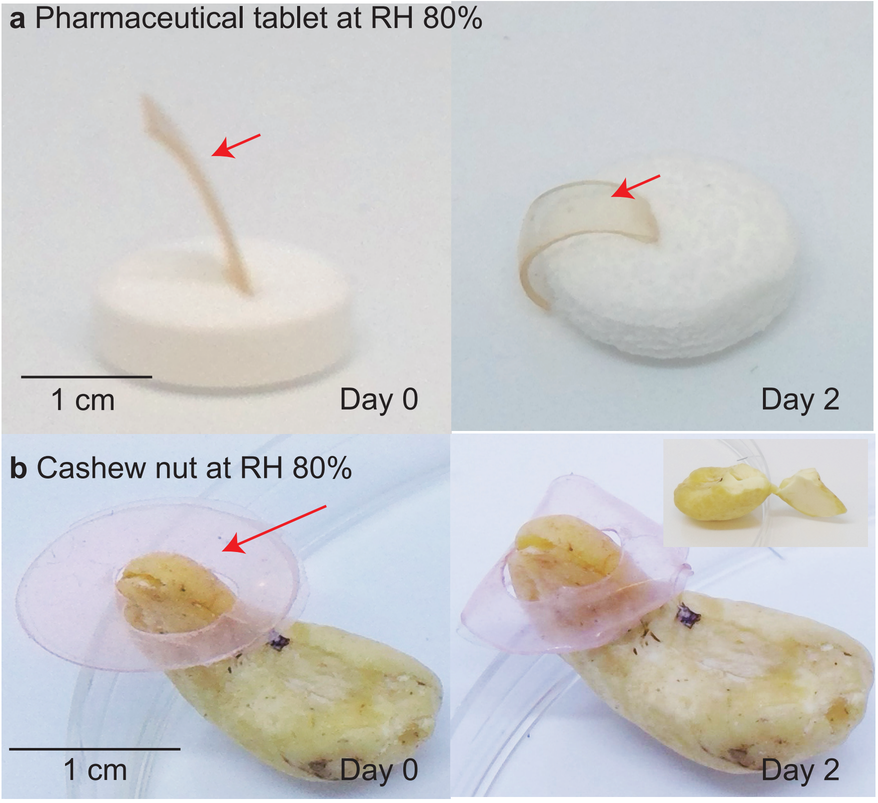

Figure S5. Two EHI applications, i.e., a pharmaceutical tablet (a) and a cashew nut (b) under 80% RH.

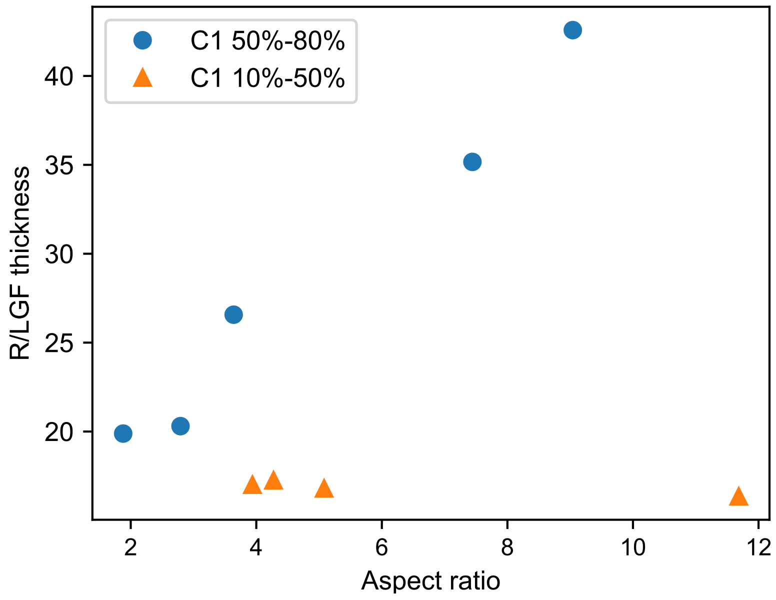

Figure S6. Normalized curvature of C1 as aspect ratio.

Table S1 Specific decomposition temperatures of various EHI films

Sample	Stage I Dehydration of H_2O			Stage II Glycerol Decomposition			Stage III Casein decompostion			Residue sample @ 700 °C
	$T_{initial}$	T_{max}	T_{final}	$T_{initial}$	T_{max}	T_{final}	$T_{initial}$	T_{max}	T_{final}	
Sodium caseinate	100	160	195		-		270	325	370	23.8%
3% glycerol	60	105	135	195	265	285	290	325	360	15.7 %
14% glycerol	55	85	110	200	250	290	290	330	360	9.0 %
3% separated film		82			250			325		13.4%
14% separated film		78			252			325		13.9 %

Movie S1: The curvature development of the indicator attached to a urine strip.
